# Social value orientation modulates fairness decision-making in empathic contexts: evidence from event-related potentials and neural oscillations

**DOI:** 10.1093/scan/nsag003

**Published:** 2026-01-28

**Authors:** Jiwen Chen, Rongrong Chen, Xinmu Hu, Yu Zhang, Xiaoqin Mai

**Affiliations:** Department of Psychology, Renmin University of China, Beijing, 100872, China; Research Center for Social Psychological Science and Engineering, Renmin University of China, Beijing, 100872, China; Department of Psychology, Renmin University of China, Beijing, 100872, China; School of Education Science, Huangshan University, Huangshan, 245021, China; Department of Psychology, Renmin University of China, Beijing, 100872, China; Department of Psychology, Renmin University of China, Beijing, 100872, China; Research Center for Social Psychological Science and Engineering, Renmin University of China, Beijing, 100872, China

**Keywords:** fairness decision-making, empathic concern, social value orientation, ERP

## Abstract

Fairness is essential for balancing interests and mitigating social conflict. People’s rejection of unfairness is influenced by contextual factors (e.g. empathic concern) and individual traits including social value orientation (SVO). This study examines how individuals with different SVOs make trade-offs between empathic concern and fairness without involving their own interests. Participants played a modified ultimatum game, in which they made decisions on behalf of either a beneficiary of a public welfare project (empathy) or a stranger (non-empathy), choosing whether to accept or reject allocation offers. Results showed that empathy increased participants’ tolerance for unfair offers, particularly among prosocials, who accepted more disadvantageous offers than proselfs did. EEG results showed that proselfs exhibited reduced N1 amplitudes in empathic conditions, reflecting attentional avoidance. Moreover, in the empathy condition, an unfairness-related MFN was observed only in proselfs. The absence of this typical MFN response in prosocials provides neural evidence for their active downregulation of unfairness aversion to prioritize the interests of empathic targets. In addition, prosocials showed stronger parietal-occipital alpha suppression and reduced P3 amplitude in empathy contexts, indicating heightened attentional arousal and greater allocation of cognitive resources. These findings highlight the crucial role of empathic concern and SVO in fairness decision-making.

## Introduction

Fairness decisions often conflict with empathic concerns for vulnerable populations, such as addressing the immediate needs of underfunded schools or vulnerable populations. In such cases, decision-making becomes particularly complex, as competing priorities must be carefully weighed. The balance between empathic concern for others and adherence to fairness principle is influenced by individual differences, particularly social value orientations (SVOs). Prosocial individuals are more concerned about others’ welfare, potentially altering their fairness decisions in empathic contexts (Hu et al. 2017). This study seeks to delve into how SVOs impact fairness decision-making, specifically in empathic contexts, using a modified ultimatum game (UG). We aim to uncover the neural mechanisms underlying how individuals with different SVOs navigate the tension between fairness and empathy, with implications for public policy and societal justice.

### Fairness decision-making and the ultimatum game

Fairness decision-making is often studied using the UG. In this game, a proposer splits money while a responder accepts or rejects the offer (rejection means neither receives anything). Unfair offers include both disadvantageous inequality (DI; unfavourable to responders) and advantageous inequality (AI; favourable to responders). The rejection of unfair offers demonstrates fairness considerations (Wu et al. 2013, Feng et al. 2019), consistent with dual-system theory which posits that fairness decisions involve both intuitive emotional processes and controlled rational processes that suppress selfish motives (Evans 2003, Lieberman 2007).

### Fairness decision-making in empathic contexts

Empathy motivates individuals to help those in distress (Batson et al. 2007). Research by He et al. (2022) found that in the UG, responders accept more disadvantageous offers and fewer advantageous offers when proposers are empathic targets, indicating self-sacrifice for empathic targets. Third-party punishment studies show harsher penalties for unfair proposers when recipients evoke empathy (Pfattheicher et al. 2019). Furthermore, individuals with high empathy tend to compensate victims, whereas those with low empathy are more inclined to punish perpetrators (Leliveld et al. 2012). These findings suggest that empathic concern modulates fairness decision, promoting altruism and stricter punishment of unfairness.

### Differences in fairness decision-making among individuals with different SVOs

Fairness decision-making is also influenced by personal traits, such as SVOs, with prosocial individuals showing greater concern for others’ welfare compared to proself individuals (Van Lange 1999, Hu et al. 2017). Prosocials demonstrate stronger fairness adherence in the UG, rejecting unfair offers due to greater inequity aversion, while proselfs are more focused on personal gains and are more likely to accept offers that benefit (Fehr and Fischbacher 2003, Hu and Mai 2021). Neuroimaging evidence reveals that prosocials exhibit heightened amygdala activation to unfair offers (Haruno and Frith 2010), corroborating their behavioural sensitivity to inequity. These SVO-related differences set the stage for considering how empathy may differentially shape fairness decisions as a function of SVO.

Crucially, SVO may determine the baseline weighting of others’ outcomes in one’s utility, while empathy makes a target’s welfare situationally more salient (Karagonlar and Kuhlman 2013). For prosocials, empathy is goal-congruent and should shift decision thresholds towards more other-regarding choices. For proselfs, empathy cues are less motivationally relevant and may even compete with gain maximization, leading to attenuated or distinct adjustments.

### Neural processing of fairness decision-making in empathetic contexts

Event-related potential (ERP) studies have identified several neural components, N1, medial frontal negativity (MFN), and P3, associated with fairness processing that can be modulated by empathy (Cui et al. 2019, Hu and Mai 2021, He et al. 2022). The N1 is a well-established neural marker of early attentional processing (Herrmann and Knight 2001, Brignani et al. 2009). Previous research has linked empathic concern to the modulation of these early attentional processes during fairness decision-making. For instance, He et al. (2022) found that when proposers were portrayed as empathy-eliciting targets (e.g. left-behind children) compared to neutral targets (e.g. ordinary students), the empathy condition evoked more negative N1 amplitudes, indicating enhanced visual attention. This aligns with broader evidence from pain-empathy studies, which shows that empathic contexts can shape the allocation of early attentional resources (Hartmann et al. 2021, Pan et al. 2023).

The MFN reflects rapid affective evaluation, with its increased amplitude in response to unfair offers—known as the “unfairness effect”—signalling a negative emotional reaction or aversive response to perceived unfairness (Gehring and Willoughby 2002, Yu et al. 2007, Cui et al. 2019). Empathic concern modulates this MFN response during fairness decisions. Specifically, He et al. (2022) observed that unfair offers from empathy-eliciting proposers (e.g. left-behind children) evoked larger MFN amplitudes, indicating a heightened negative emotional response.

The P3 reflects the increased cognitive load associated with evaluation (Wu et al. 2011, Luo et al. 2014). Research has shown that the P3 is influenced by reward and personal interest considerations, suggesting that it may not be modulated by empathy in self-interested contexts (He et al. 2022). Further research is needed to examine potential empathy effects in non-self-interested contexts. Additionally, alpha oscillations reflect attentional engagement (Lange et al. 2013, Marshall et al. 2015), and investigating this component helps explore the role of empathy in fairness decision-making mechanisms.

### Present study

While prior research has established the distinct roles of empathy (He et al. 2022) and SVOs (Hu and Mai 2021) in fairness decisions, their interaction remains poorly understood. Specifically, it is unclear how individual differences in SVOs modulate fairness decisions in empathic contexts devoid of self-interest, particularly under advantageous inequality. Furthermore, the neural mechanisms underpinning this interaction remain unexplored. Elucidating this interaction and its neural dynamics is crucial for a comprehensive understanding of how empathy shapes fairness decisions and for distinguishing early affective responses from subsequent controlled cognitive processing.

This study aims to examine differences in fairness decision-making behaviours among individuals with different SVOs in empathic contexts, focusing on the neural mechanisms underlying the trade-off between fairness and empathic concern. The study extends prior research in two primary ways. First, by examining how empathy towards vulnerable individuals interacts with trait SVO in third-party decision-making scenarios that lack direct self-payoff, it tests whether the established tendency for empathy to promote tolerance of unfair behaviour is a general phenomenon or is predominantly characteristic of prosocial individuals. Furthermore, it advances SVO theory by delineating how stable, other-oriented preferences operate in purely prosocial settings when fairness norms conflict with the needs of vulnerable others. Second, by integrating behavioural measures with ERP components and neural oscillations, the study offers a temporally detailed, process-oriented explanation of how SVO and empathy jointly shape fairness decisions. This approach allows for the dissociation between early-stage attentional and affective processing and later-stage controlled evaluation, thereby refining current neurocognitive models of social decision-making that incorporate fairness norms, empathic concern, and trait-based individual differences.

We use a modified UG task in which participants act as third-party observers, making decisions on behalf of recipients who are either empathic or non-empathic targets. Previous research by He et al. (2022) showed that participants were more likely to accept unfair offers when the proposer was an empathic target, suggesting a prioritization of the empathic proposer’s interests over strict fairness. Additionally, Karagonlar and Kuhlman (2013) found that when accepting unfair offers, prosocials develop more positive cognitions than proselfs regarding both the proposer and the unfair offer, possibly through cognitive reappraisal, suggesting that they manage emotional responses to unfairness more effectively (Gross 2002, Goldin et al. 2008). Based on these findings, we propose the following hypotheses:

**Hypothesis 1:** Compared to the non-empathy condition, participants will more frequently accept allocation proposals that favour empathic targets, particularly advantageous inequality offers, which are most beneficial to empathic targets.

**Hypothesis 2:** Prosocials will be more concerned about the interests of empathic targets, especially when there is a conflict between a strong aversion to unfairness and the interests of the target (e.g. in disadvantageous inequality offers). Prosocials are expected to exhibit higher acceptance rates than proselfs to ensure minimal benefits for empathic targets. This heightened concern might be reflected in decreased alpha oscillations and potentially in N1 amplitude.

**Hypothesis 3:** Empathy is cognitively demanding, and proselfs may exhibit early attentional avoidance of empathic concerns compared to prosocials, leading to smaller N1 amplitudes in empathic contexts. In contrast, prosocials may allocate greater cognitive resources and exhibit heightened attentional arousal in empathic contexts, resulting in reduced P3 amplitudes and increased alpha suppression.

**Hypothesis 4:** The MFN components are influenced by emotional processes. Prosocials, who are better at using cognitive reappraisal, will show attenuated unfairness effects in MFN under empathic contexts, while proselfs will continue to exhibit these effects even in empathic contexts.

## Methods

### Participants

Sixty-seven undergraduate and graduate students (all female) from Renmin University of China participated in this study. All participants were right-handed, had normal or corrected-to-normal vision, and reported no history of psychiatric or neurological illness. Due to excessive movement artifacts, nine participants were excluded from the analysis, leaving a final sample of 58 participants. The final cohort comprised 31 prosocial individuals (mean age = 20.03 ± 1.80 years) and 27 proself individuals (mean age = 20.67 ± 2.83 years). All participants signed the informed consent form and received 90 Chinese yuan for their participation. This study was approved by the Institutional Review Board of the Department of Psychology at Renmin University of China.

### Materials and procedure

Prior to the main experiment, participants completed the online SVO Slider Measure (Murphy et al. 2011). On this six-item measure, participants were asked to choose between several self-other payoff combinations. These decisions yield coordinates in a two-dimensional space defined by the participant’s own payoff and the other’s payoff, from which an individual SVO angle is computed. Following the standard classification procedure (Murphy et al. 2011), an SVO angle of 22.45° served as the categorical boundary: participants with scores below this threshold were classified as proself, while those with scores above it was classified as prosocial.

On arrival at the laboratory, they filled out the paper version of the Interpersonal Reactivity Index (IRI) scale assessing trait empathy, and then performed the third-party decision task while EEGs were recorded. Participants’ state empathy was manipulated and checked at the beginning of each block of the third-party decision task. Details of all scales used in the study are provided in the Supplementary Material.

#### Manipulation and assessment of state empathy

At the beginning of each block of the third-party decision task, the participant’s empathy state was manipulated and checked. Each participant performed the task six times, making decisions on behalf of six different beneficiaries: three in need of help (empathy condition) and three not in need (non-empathy condition). In the empathy condition, participants were required to select one beneficiary at a time from four public welfare projects and make a decision for him/her in the third-party decision task; these projects sourced from a widely recognized platform for public welfare donations, Alipay’s official public welfare platform (https://love.alipay.com/donate/index.htm). In the non-empathy condition, participants were required to choose one at a time from four unknown phone numbers belonging to individuals who had previously participated in unrelated studies and were thus unknown to the current participants, and to make decisions for the owner of that phone number.

#### Performing the third-party decision task

Participants were seated comfortably in front of a computer screen in an electrically isolated room to complete the third-party decision task adapted from the UG. The task involved three roles: proposer, recipient, and decision-maker. The proposers were individuals’ unknown to the participants; the recipients were individuals either in need of help (empathy condition) or not in need of help (non-empathy condition), selected by the participants during the empathy manipulation phase. The participants themselves played the role of decision-maker. In each round of the task, the proposer and the recipient were to divide a total of 10 Chinese Yuan, with the proposer making the allocation proposal. The decision-maker (the participant) then decided whether to accept or reject the proposal on behalf of the recipient. If the decision-maker accepted the proposal, both the proposer and the recipient received money according to the proposal; if rejected, neither party received any money.

**Figure 1.**
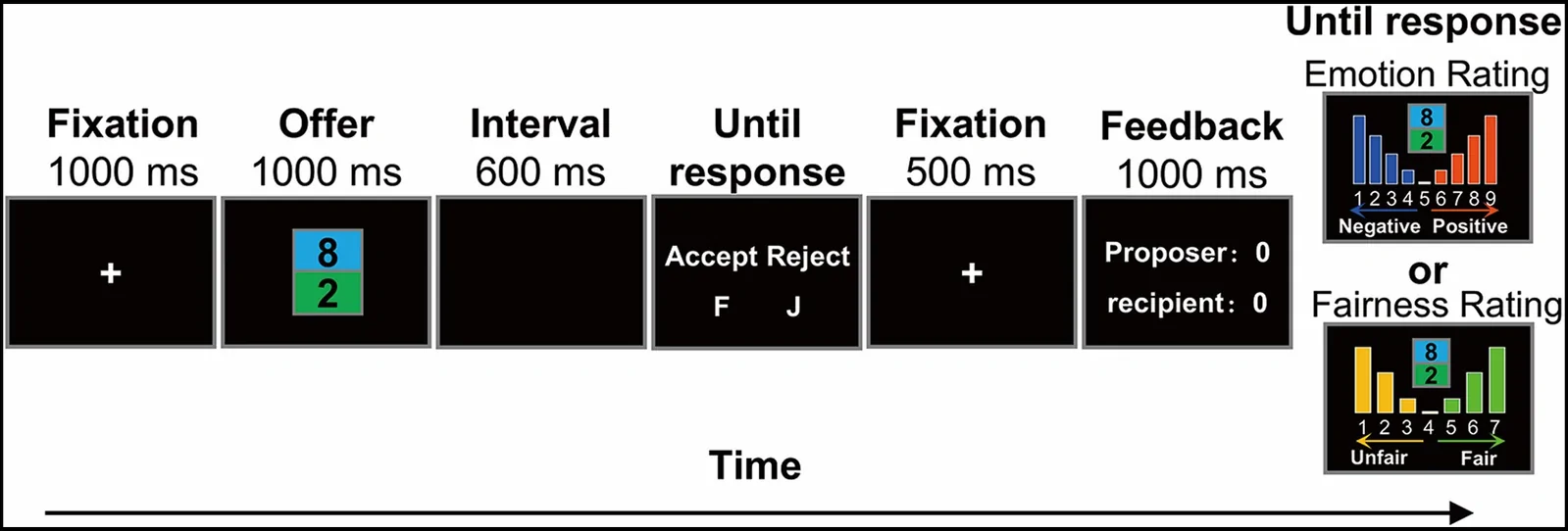
Illustration of a single trial of the third-party decision task.

Figure 1 illustrates the time course of stimulus presentation in the third-party decision task. Each trial began with a white fixation cross presented on a black background for 1000 ms. Then, the offer screen was presented for 1000 ms, depicting a distributive outcome of 10 yuan between the proposer and the recipient. The numbers in the blue square at the top of the screen represented the amount allocated by the proposer to himself/herself, and the numbers in the green square at the bottom of the screen represented the amount allocated to the recipient. After a 600 ms black screen, the text “Accept” and “Reject” appeared on the screen. Participants made a choice on behalf of the recipient by pressing the F or J key on the keyboard with their left or right index finger. Pressing the F key represented accepting the offer and pressing the J key represented rejecting the offer. After an interval of 500 ms, feedback on the offer was presented for 1000 ms. If participants accepted the offer, the money was split between the proposer and recipient as proposed. If rejected, the proposer and recipient received nothing. In addition, participants rated their emotional reaction to the current offer on a 9-point scale (1 = *extremely negative*, 9 = *extremely positive*), or rated the perceived fairness of the current offer on a 7-point scale (1 = *extremely unfair*, 7 = *extremely fair*) (Hu and Mai 2021). For each offer within each block, the rating modality (emotion/fairness) was randomly assigned and recorded once.

The entire task was divided into six blocks (three empathy blocks, three non-empathy blocks) presented randomly, with brief breaks between blocks. Participants’ empathy state was manipulated and assessed before the onset of each block. Each block consisted of 60 trials: 18 fair trials (six each of 5-5, 4-6, and 6-4 offers), 18 disadvantageous inequality trials (nine each of 9-1 and 8-2 offers), 18 advantageous inequality trials (nine each of 1-9 and 2-8 offers), and six filler trials (three each of 3-7 and 7-3 offers). In total, there were 54 trials for each of the six conditions: Equality (EQ), disadvantageous inequality, and advantageous inequality offers in empathy and non-empathy conditions. Unbeknownst to the participants, all allocation offers were randomly presented, computer-generated, and not proposed by actual individuals. Before the formal task, all participants were familiarized with the procedure through five practice trials. Stimulus presentation and behavioural data acquisition were conducted using E-Prime 3.0 software (PST, Inc., Pittsburgh, PA, USA).

### EEG recording and preprocessing

EEG data were recorded from 64 cap-mounted tin electrodes arranged according to the 10/20 international placement system (Neuroscan Inc., Herndon, VA, USA), with an online reference to the left mastoid and an offline re-reference to the averaged mastoid electrodes. Vertical electrooculography (EOG) was recorded from electrodes placed above and below the left eye, while horizontal EOG was recorded from electrodes placed 1.5 cm laterally to the outer canthi of both eyes. Signals were amplified using a 0.01–100 Hz band-pass filter and continuously sampled at 500 Hz per channel. Inter-electrode impedances were maintained below 5 kΩ. Offline analysis of EEG data was performed using the eeglab2023.1 toolbox of Matlab (2023a). The EEG data were low-pass filtered below 30 Hz (24 dB/oct). Epochs containing artifacts exceeding ±75 µV were excluded from subsequent analyses. Independent component analysis (ICA) was used to eliminate artifacts such as eye movements, blinks, and electromyographic noise from all electrode sites. On average, no more than five ICA components were removed per participant.

### ERP analysis

The EEG data were segmented into epochs time-locked to the onset of the offer presentation, beginning 200 ms before the offer onset and continuing for 1000 ms. The epochs were baseline-corrected according to the 200 ms pre-offer period, and were then averaged separately for each condition of each participant. Based on previous studies (Hu and Mai 2021, He et al. 2022) and inspection of the grand-averaged waveforms, N1, MFN, and P3 were quantified as the minimum peak amplitude within 100–200 ms, the mean amplitude within 290–390 ms, and the mean amplitude within 350–550 ms after the onset of offer presentation, respectively. Based on topographies of each ERP components, the N1 and MFN were measured at frontal-central electrode Fz and the P3 was measured at centro-parietal electrode Pz.

To address potential overlaps between MFN and P3 in the ERP waveforms, principal component analysis (PCA) was employed to decompose the ERP components (Foti et al. 2011). The time-domain PCA was conducted using the Evoked ERP/EPO toolbox in Matlab (Zhang et al. 2020). Three PCA factors, PCA-N1, PCA-MFN, and PCA-P3, were identified. Further analysis showed that the PCA results were consistent with traditional ERP results. A detailed PCA analysis and its results can be found in the Supplementary Material.

### Time-frequency analysis

Time-frequency distributions (TFDs) of EEG trials were estimated using a windowed Fourier transform with a fixed 200 ms Hanning window. It yielded, for each trial, a complex time-frequency estimate F(*t*, *f*    ) at each time-frequency point (*t*, *f     *), extending from prestimulus 1000 ms to post-stimulus 1000 ms in the time domain, and from 1 to 30 Hz in the frequency domain. The resulting spectrogram, P(*t*, *f*   ) = |F(*t*, *f*     )|^2^, represents the signal magnitude as a joint function of time and frequency at each time-frequency point. We focused on the α-oscillations following offer stimulus onset. Based on the previous studies on α-oscillations, we calculated the average oscillation power of alpha oscillations (8–13 Hz) during a time window of 300 ms to 600 ms following offer stimulus onset. A mean amplitude-based baseline-correction procedure was applied within the -450 and -50 ms relative to the onset of offer. Scalp topographies of alpha oscillation were computed by spline interpolation. In these analyses, we focused on the parieto-occipital electrodes PO3, POz, PO4.

### Statistical analyses

Acceptance rates (ARs), reaction times (RTs), emotional ratings, fairness ratings, and ERP data were each subjected to a 2 (SVO type: prosocial, proself) × 2 (Empathy induction: empathy, non-empathy) × 3 (Fairness: disadvantageous inequality, equality, advantageous inequality) mixed three-way repeated measures analysis of variance (ANOVA), with SVO type as a between-subjects variable and empathy induction and fairness as within-subjects variables. The significance level was set at 0.05 for all analyses. Greenhouse-Geisser correction was performed to account for sphericity violations, where appropriate. Post-hoc analyses with Bonferroni correction were conducted for significant main effects.

## Results

### Assessment of trait empathy and state empathy

There was no significant difference in trait empathy between the proself and prosocial groups. Manipulation checks, implemented as comparisons of state empathy ratings between the empathy and non-empathy conditions, confirmed the effectiveness of the empathy induction (empathy vs. non-empathy: 5.93 ± 0.11 vs. 3.13 ± 0.18, *P *< .001) (see Supplementary Material for details).

### Behavioural results and subjective ratings

**Figure 2.**
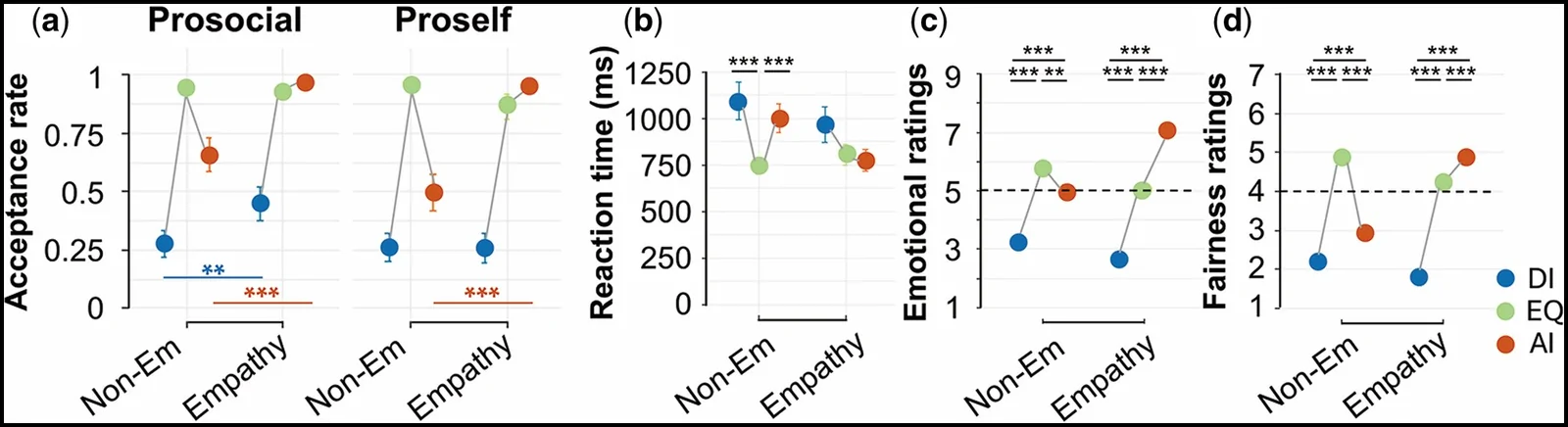
Interaction effect among SVO type, empathy induction, and fairness on acceptance rate (a). Interaction effect between empathy induction and fairness on reaction time (b), emotional ratings (c), and fairness ratings (d). Error bars indicate standard errors (SE). Non-Em: non-empathy; DI: disadvantageous inequality; EQ: equality; AI: advantageous inequality. **P < .01, ***P < .001.

Behavioural results showed that empathy increased participants’ ARs for unfair offers, particularly among prosocials, who accepted more disadvantageous offers than proselfs (Fig. 2a). Reaction times were faster for equal offers without empathy, but this difference disappeared under empathy (Fig. 2b). Subjective ratings mirrored behavioural findings: emotional and fairness ratings were highest for equal offers without empathy, but shifted towards advantageous inequality offers with empathy (Fig. 2c and d; see Supplementary Material for details).

### ERP results

#### N1

**Figure 3.**
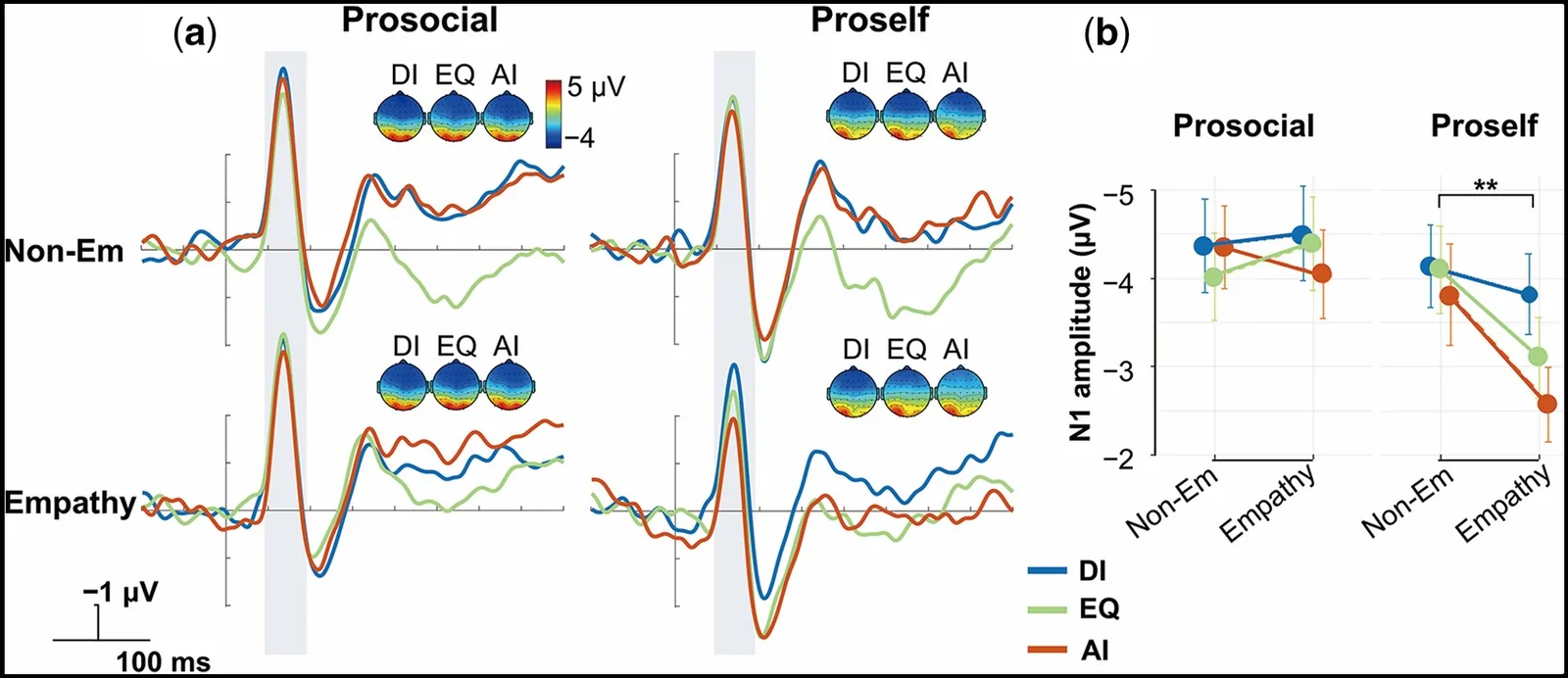
(a) Grand-average ERP waveforms from the Fz electrode and scalp topographies of the N1. The shaded portion indicates the time window of the N1 (100–200 ms) used for statistical analysis. (b) Mean value of N1 peak amplitude for each condition. Error bars indicate standard errors (SE). DI: disadvantageous inequality; EQ: equality; AI: advantageous inequality; Non-Em: non-empathy. **P < .01.

An ANOVA on N1 amplitude revealed significant main effects of empathy induction, *F*(1,56) = 4.54, *P *= .037, η_p_^2^ = 0.08, and fairness, *F*(2,112) = 8.81, *P *= .004, η_p_^2^ = 0.14. Significant two-way interactions were observed between empathy induction and SVO type, *F*(1,56) = 6.31, *P *= .015, η_p_^2^ = 0.1, and between empathy induction and fairness, *F*(2,112) = 4.09, *P *= .048, η_p_^2^ = 0.07. To further explore the interaction between empathy induction and SVO type, simple effects analyses were conducted. The results (Fig. 3) showed that prosocial individuals exhibited no significant difference in N1 amplitude between empathy and non-empathy conditions (*P *≥ .1). In contrast, proself individuals demonstrated significantly reduced N1 negativity under empathy induction (−3.16 μV) compared to the non-empathy condition (−4.01 μV), *P *= .002.

**Figure 4.**
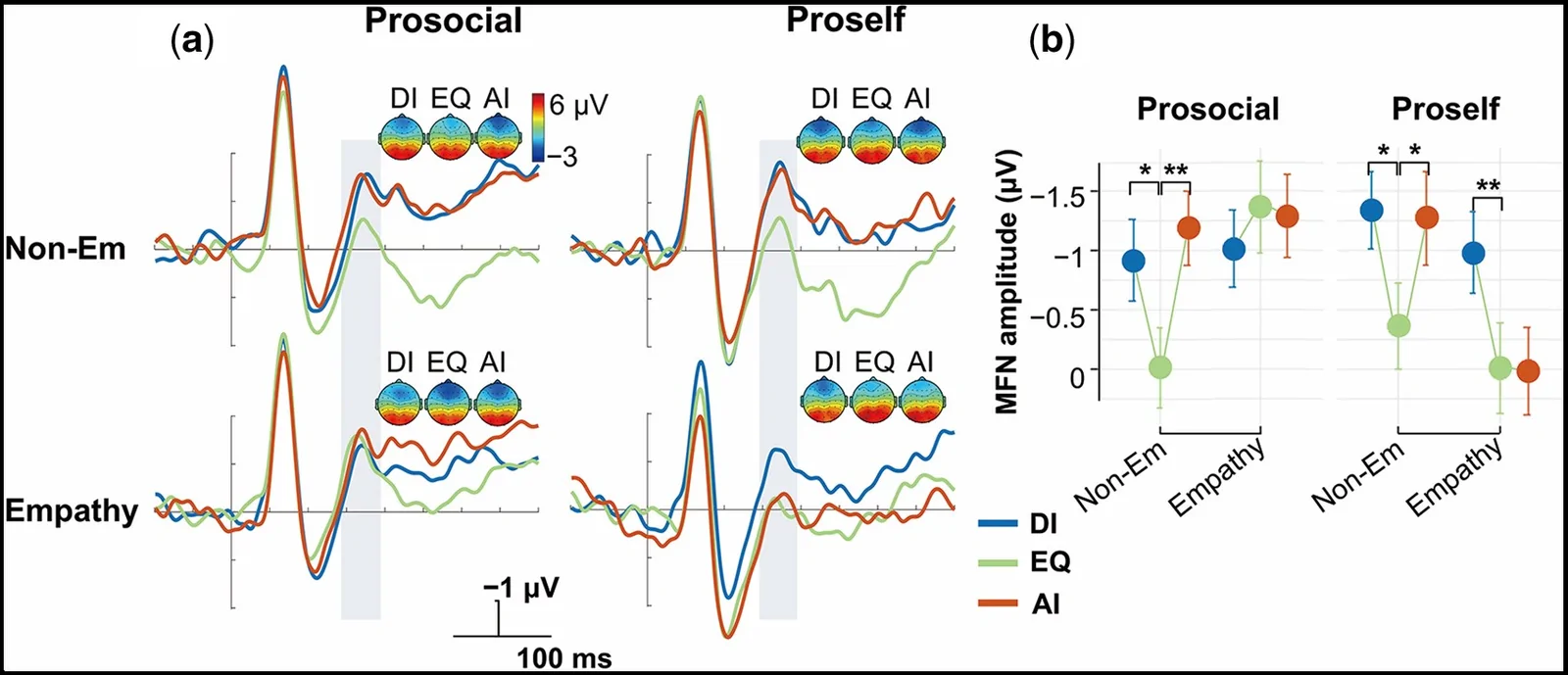
(a) Grand-average ERP waveforms from the Fz electrode and scalp topographies of the MFN. The shaded portion indicates the time window of the MFN (290–390 ms) used for statistical analysis. (b) Mean value of MFN amplitude for each condition. Error bars indicate standard errors (SE). DI: disadvantageous inequality; EQ: equality; AI: advantageous inequality; Non-Em: non-empathy. *P < .05, **P < .01.

#### Medial frontal negativity

An ANOVA on MFN amplitude revealed a significant main effect of fairness, *F*(2,112) = 9.10, *P *= .004, η_p_^2^ = 0.14, as well as significant two-way interactions between empathy induction and SVO type, *F*(1,56) = 5.20, *P *= .026, η_p_^2^ = 0.09, between fairness and SVO type, *F*(2,112) = 4.01, *P *= .05 η_p_^2^ = 0.07, and between empathy induction and fairness, *F*(2,112) = 5.11, *P *= .028, η_p_^2^ = 0.08. The three-way interaction (SVO × empathy induction × fairness) was not significant, *F*(2,112) = 2.56, *P *= .116, η_p_^2^ = 0.044.

Given our a priori interest in SVO, we additionally conducted exploratory within-group empathy induction × fairness simple-effects analyses (Fig. 4). In the non-empathy condition, unequal offers (both disadvantageous and advantageous inequality) elicited significantly more negative MFN amplitudes than equal offers in both groups. This was shown by comparing equality with disadvantageous and advantageous inequality in both the prosocial (−0.02 vs. −0.92 μV, *P *= .019; −0.02 vs. −1.19 μV, *P *= .006) and proself groups (−0.37 vs. −1.35 μV, *P *= .024; −0.37 vs. −1.28 μV, *P *= .045). In the empathy condition, the proself group displayed significantly enhanced MFN for disadvantageous inequality offers (−0.98 μV) compared to both equality (−0.004 μV, *P *= .01) and advantageous inequality offers (0.02 μV, *P *= .02), whereas no differences was observed among the three offer types for the prosocial group.

#### P3

**Figure 5.**
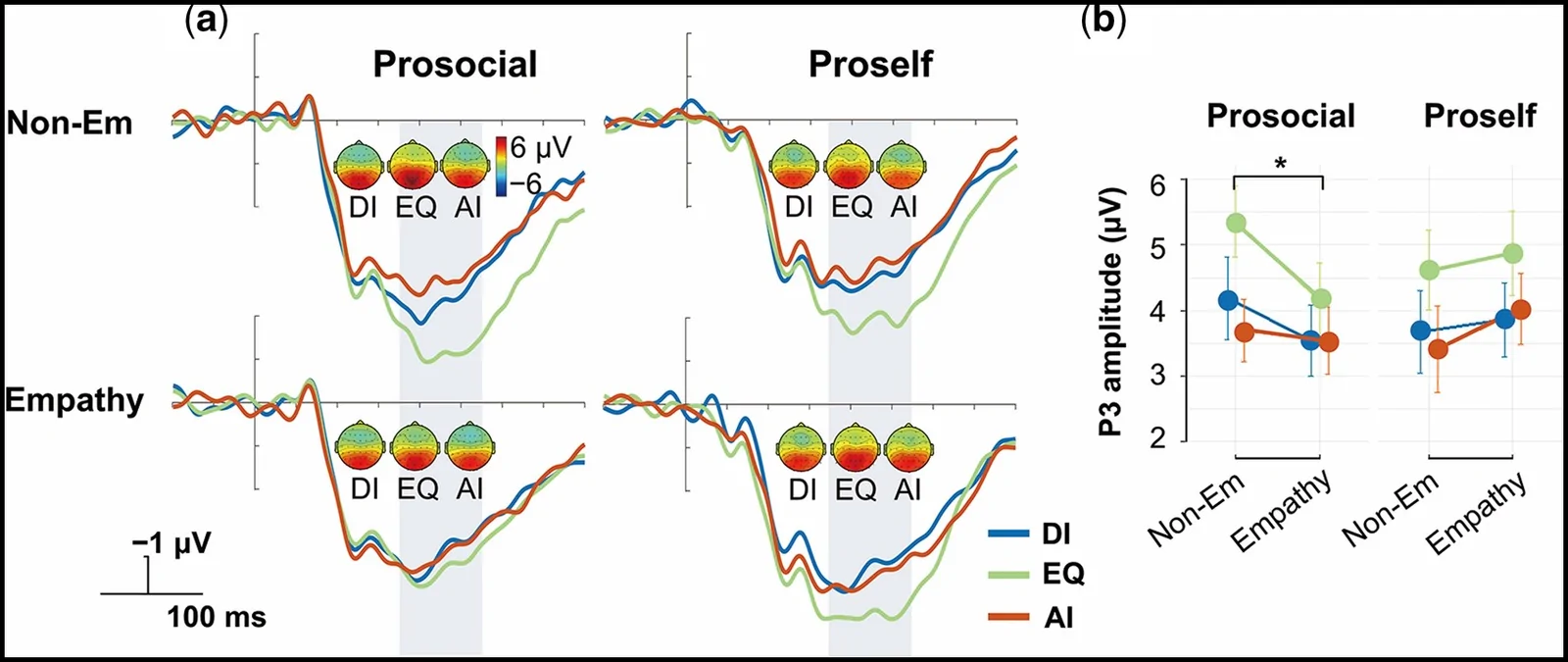
(a) Grand-average ERP waveforms from the Pz electrode and scalp topographies of the P3. The shaded portion indicates the time window of the P3 (350–550 ms) used for statistical analysis. (b) Mean value of P3 amplitude for each condition. Error bars indicate standard errors (SE). DI: disadvantageous inequality; EQ: equality; AI: advantageous inequality; Non-Em: non-empathy. *P < .05.

An ANOVA on P3 amplitude revealed a significant main effect of fairness, *F*(2,112) = 29.40, *P *< .001, η_p_^2^ = 0.34, and a significant interaction between empathy induction and SVO type, *F*(1,56) = 5.18, *P *= .027, η_p_^2^ = 0.09. To further explore the interaction between empathy induction and SVO type, simple effects analyses were conducted. The results (Fig. 5) showed that proself individuals did not show a significant difference in P3 amplitude between empathy and non-empathy conditions (*P *≥ .1). In contrast, prosocial individuals showed a significantly reduced P3 amplitude under empathy induction (3.84 μV) compared to the non-empathy condition (4.46 μV), *P *= .048.

### Time-frequency results

#### Alpha oscillation

**Figure 6.**
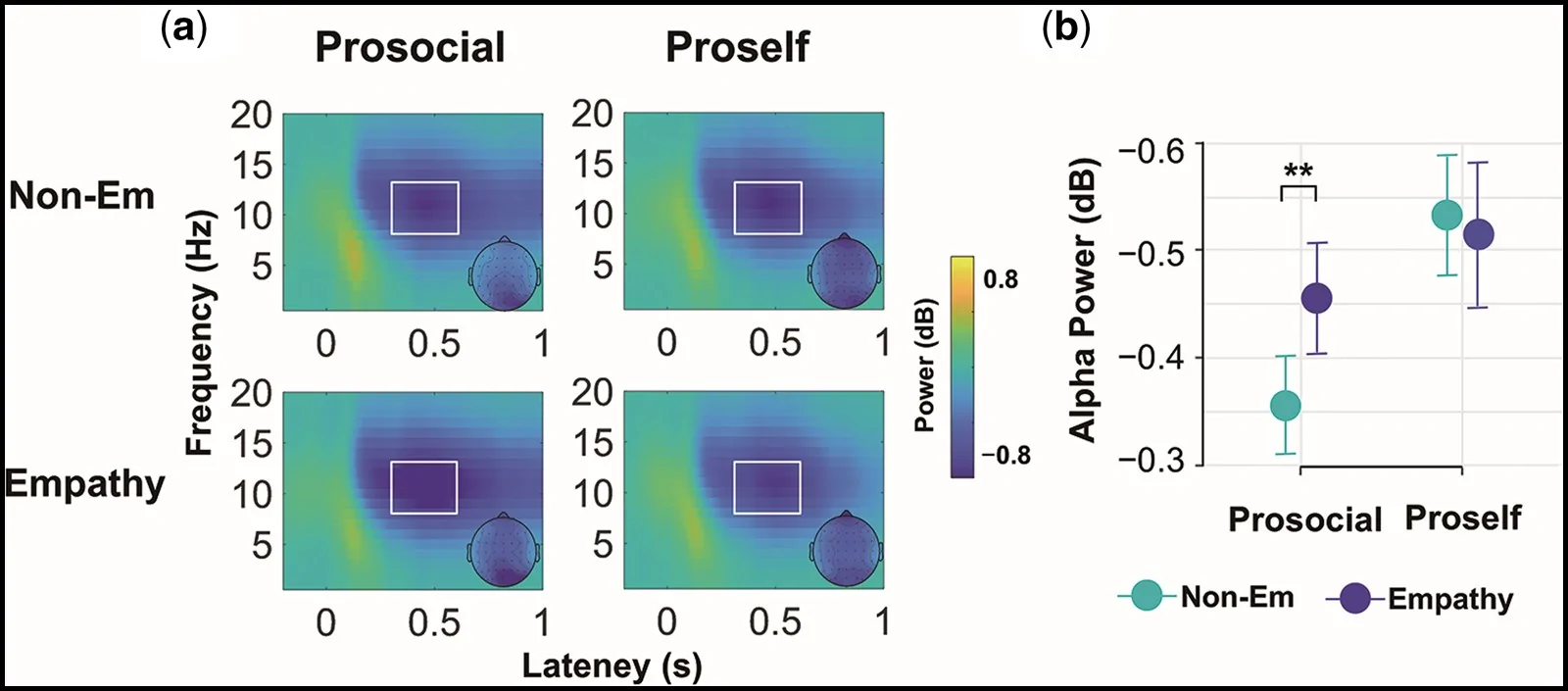
(a) Alpha oscillation spectra and topographic mapping of alpha power distribution in the parieto-occipital region (PO3, POz, PO4) after stimulation (Window: 300–600 ms). (b) Mean value of alpha power for each group. Error bars indicate standard errors (SE). Non-Em: non-empathy. **P < .01.

An ANOVA on the alpha oscillation revealed a significant interaction effect between SVO and empathy induction, *F*(1,56) = 5.92, *P *= .018, η_p_^2^ = 0.1. A simple effects analysis (Fig. 6) showed that for the prosocial group, alpha suppression was significantly stronger in the empathy condition than in the non-empathy condition (−0.45 vs. −0.35 dB, *P *= .004). In contrast, for the proself group, no difference was observed between the empathy and the non-empathy conditions (*P *≥ .1).

**Figure 7.**
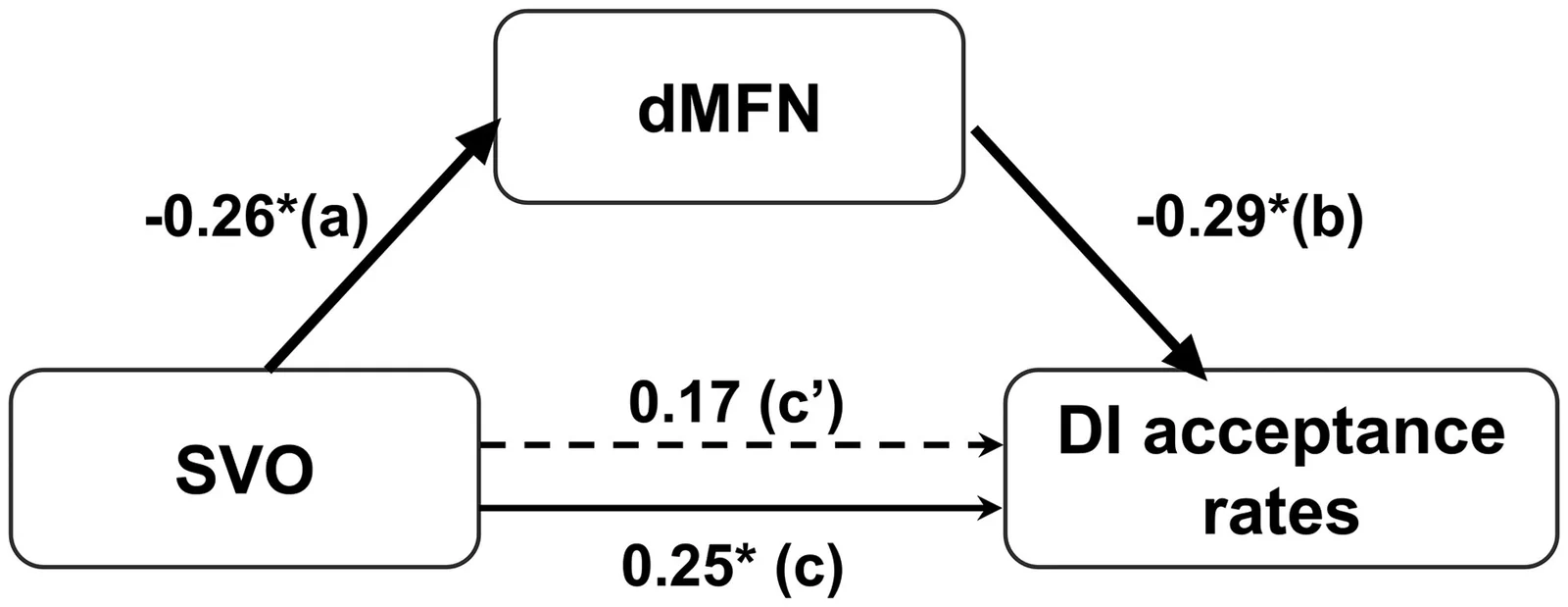
Mediation effect: the effect of SVO on acceptance rates for disadvantageous inequality (DI) offers was significantly reduced when dMFN was included in the SEM. Standardized estimates are shown. *Proselfs* are encoded as 0, *prosocials* as 1. *P < .05.

### Mediation analysis results

Pearson correlation analyses were conducted separately for the empathy and non-empathy conditions to examine the associations among individuals’ SVO, neural indices, and acceptance rates under unfair conditions (i.e. both advantageous and disadvantageous inequality). Comprehensive correlation results are reported in the Supplementary Material. In the empathy condition, SVO, dMFN (defined as the MFN amplitude for fair offers minus that for disadvantageous unfair offers), and the acceptance rate for disadvantageous inequality were all pairwise correlated. Since these conditions met the conditions for mediation analysis, we tested an indirect effect through dMFN. In the non-empathy condition, the data did not support a mediation analysis, and thus none was conducted.

The structural equation model (SEM) indicated a significant positive direct effect of SVOs on acceptance rates ( *β*  =  0.25, *P *= .03, Fig. 7). Upon inclusion of dMFN, SVOs demonstrated a significant negative effect on dMFN (* β* = −0.26, *P *= .028), while dMFN exerted a significant negative effect on acceptance rates (* β* = −0.29, *P *= .025). Crucially, the direct effect of SVOs on acceptance rates became non-significant, indicating full mediation by dMFN. Bootstrapping results indicated that this mediation effect was significantly different from zero with 95% confidence (number of bootstrap resamples = 5000; mediation effect = 0.076, confidence intervals = 0.001 to 0.152). The confidence intervals for the indirect effect were bias-corrected (Preacher and Hayes 2008).

## Discussion

This study examines how individuals with different SVOs make trade-offs between empathic concern and fairness without involving their own interests. Behaviourally, empathy significantly influences the acceptance of unfair offers, with differences between SVO groups primarily observed in the disadvantageous inequality condition. EEG results revealed that empathy induction prompted reduced N1 amplitudes in proselfs, who also showed a significant MFN response to disadvantageous inequality offers. In contrast, prosocials exhibited stronger alpha suppression and reduced P3 amplitudes. Furthermore, a mediation analysis demonstrated that individual differences in dMFN statistically accounted for part of the relationship between SVOs and acceptance rates under disadvantageous inequality.

### The impact of empathic contexts on fairness decision-making

The results indicate that empathy plays a key role in fairness-related decision-making. In the non-empathy context, participants showed higher acceptance rates, fairness ratings, and emotional ratings for equality than for both advantageous and disadvantageous inequality offers, indicating a preference for equality over unfairness. RTs were also faster for equality offers, consistent with an automatic bias towards fairness.

In the empathic context, however, the relationship between fairness and decision-making becomes more complex. Participants exhibited higher ARs, emotional and fairness ratings for advantageous inequality offers that allocated more resources to the recipient with whom they empathized, suggesting that decisions in empathic contexts deviate from strict distributive fairness, but are perceived as fairer when viewed from social or emotional perspectives. Emotional experiences and fairness perceptions are important factors that drive individuals to engage in irrational behaviours that deviate from the principle of maximizing personal gain (Luo et al. 2014, He et al. 2022). This suggests that empathy shifts the decision-making process from a purely objective evaluation of fairness to a more personalized consideration in which the welfare of the empathized individual becomes a central factor. In this context, participants were more likely to prioritize the needs or outcomes of others, even if this meant deviating from an equal distribution, consistent with the notion that empathy can promote altruistic motives (Cui et al. 2022, He et al. 2022).

### Individuals with different SVOs differ in fairness decision-making behaviour in empathic contexts

The present study found that, in the non-empathy context, both prosocials and proselfs showed similarly low ARs for disadvantageous inequality offers. However, in the empathic context, prosocials showed an increase in their ARs for these offers. This suggests that both groups have an aversion to disadvantageous inequality offers (Dawes et al. 2007, Chen and Vazsonyi 2011, Dawes et al. 2012); however, prosocials are more likely to consider the interests of empathic targets and tolerate unfairness in order to benefit them (Van Lange 1999, Bogaert et al. 2008).

According to the integrated model of social value orientation (Van Lange 1999), prosocials prioritize fairness and are more attuned to others’ needs than proselfs who focus on their own outcomes (Haruno and Frith 2010). Empathy and willingness to help not only increase prosocials’ understanding, but also motivate them to contribute, even in the face of unfair offers (Karagonlar and Kuhlman 2013). Consequently, prosocials adopt a more positive attitude towards disadvantageous inequality offers, exhibiting higher ARs than proselfs to ensure minimal benefits for empathetic targets, reflecting the notion that “something is better than nothing.”

Building on this motivational framework, the emotion regulation-self-control hypothesis (Karagonlar and Kuhlman 2013) might provide a mechanistic explanation. Prosocials demonstrate superior cognitive reappraisal skills, which enable them to reframe emotional responses during early stages of processing (Gross 2002, Goldin et al. 2008, van’t Wout et al. 2010). This regulatory capacity allows them to suppress fairness-related aversion, thereby increasing ARs to disadvantageous inequality offers when prioritizing the welfare of others. These findings advance our understanding of how empathic concern interacts with orientation-specific cognitive mechanisms to shape perceptions of fairness.

### The neural processing of SVO modulating fairness decision-making in empathic contexts

The N1 component serves as an early neurophysiological indicator of attentional allocation (Zhang et al. 2017). In this study, proself individuals exhibited smaller N1 amplitudes in the empathic context compared to the non-empathic context, whereas no such effect was observed in prosocial individuals. Empathy, a complex cognitive-affective process, demands substantial cognitive resources (Cameron 2018, Wu et al. 2020). For proself individuals, whose motivational framework prioritizes self-protection and personal gain, engaging in empathy-related processing may represent a potential cognitive resource drain. We posit that this group actively disengages from empathy when confronted with emotionally salient stimuli to conserve cognitive resources, resulting in the attenuated N1 amplitudes. Alternatively, the reduced N1 in proselfs may also reflect more efficient, less resource-demanding initial processing of stimuli in an empathic context they perceive as less motivationally relevant.

The MFN represents the middle stage of fairness processing, reflecting expectation violations and unfairness aversion (Gehring and Willoughby 2002, Cui et al. 2019). In non-empathic contexts, for both proself and prosocial groups, unfair offers elicited more negative MFN amplitudes than equality offers, suggesting violations of fairness expectations and participants’ aversion to unfairness. In empathic contexts, however, only proselfs exhibited an MFN unfairness effect. For prosocials, their heightened sense of responsibility towards empathic targets may attenuate the MFN unfairness effect. This reduction in neural sensitivity may be due to the involvement of the medial prefrontal cortex in inhibitory control and complex rule processing (Deyoung and Gray 2009, DeYoung et al. 2010). By prioritizing others’ welfare, prosocials may employ cognitive reappraisal strategies to downregulate unfairness aversion, thereby diminishing the MFN unfairness effect (Karagonlar and Kuhlman 2013). In contrast, proselfs driven by self-interest exhibited the typical MFN unfairness response. Further mediation analysis indicated that prosocials displayed a smaller MFN unfairness effect compared to proselfs, and interindividual variability in this effect statistically accounted for a portion of the association with higher acceptance rates of disadvantageous inequality offers. These findings are consistent with the view that, in empathic contexts, prosocials show reduced neural sensitivity to unfairness and tend to accept disadvantageous inequality offers more often.

The P3 is a well-established neural correlate of cognitive resource allocation, motivational salience, and higher-level information integration (Yang et al. 2015, Zhang et al. 2017). In the present study, prosocials exhibited significantly reduced P3 amplitudes in the empathic context compared to the non-empathic context, a pattern not observed among proselfs. One plausible interpretation, consistent with evidence that attenuated P3 amplitudes can reflect increased cognitive load (Chen et al. 2025), is that prosocials engage in more extensive deliberative processing during empathy-related decision-making. For instance, considerations such as whether insistence on equality overlooks a target’s predicament likely demand additional cognitive resources for information integration and emotional perspective-taking. In contrast, proselfs may base fairness judgments predominantly on self-interest; consequently, additional empathic cues may not substantially engage their evaluative resources. Alternatively, the P3 amplitude reduction could be explained by differential contextual information integration. Prosocials may more deeply integrate empathic cues with fairness norms and situational factors—a process supported by fronto-parietal evaluative systems (Wu and Hong 2022). This integrative processing could shift the focus of attentional resource allocation away from the immediate offer stimulus, thereby reducing the resources devoted to its evaluation and resulting in attenuated P3 responses.

Alpha oscillations index cortical excitability, sustained attention, and broader forms of information regulation, and dynamically vary with cognitive states (Clayton et al. 2015, Baumgarten et al. 2016, Sadaghiani and Kleinschmidt 2016, Pan et al. 2023). Reduced alpha power is typically associated with heightened cortical excitability and attentional engagement (Marshall et al. 2015). In the present study, prosocials exhibited enhanced alpha suppression during empathy induction compared to the non-empathic context, suggesting intensified attentional focus while evaluating offers for empathic targets. An alternative explanation is that empathic contexts may require prosocials to coordinate evaluative and regulatory processes across frontal-parietal networks. Such network-level interactions can be reflected in alpha-band desynchronization, indicative of increased cortical excitability and flexible information exchange. Importantly, this pattern aligns with the reduced P3 amplitudes observed in prosocials. Taken together, these findings suggest that empathic decision-making may impose greater cognitive processing demands on prosocial individuals.

### Limitations and future prospects

This study has several limitations. First, emotional responses were assessed solely via subjective reports after the decision had been made, which may introduce recall bias and obscure real-time affective dynamics. Future research should therefore integrate real-time physiological indices (e.g. heart rate variability and skin conductance) to capture objective emotional responses *during* decision-making. Second, the inclusion of only female participants limits the study’s generalizability. Female participants were selected because they typically exhibit stronger empathy, ensuring sufficient empathy induction. Additionally, ERP components are sensitive to individual differences, so a homogeneous, female-only sample was chosen to reduce between-subject variance and enhance statistical power. Subsequent studies should therefore incorporate male participants to examine gender-specific modulation of fairness decisions in empathic contexts.

## Supplementary Material

nsag003_Supplementary_Data

## Data Availability

The data supporting this study’s findings are available from the corresponding author on reasonable request.
