# Supplementary material for: Social value orientation modulates fairness decision-making in empathic contexts: evidence from event-related potentials and neural oscillations
Source: Soc Cogn Affect Neurosci. 2026 Jan 28;21(1):nsag003. doi: 10.1093/scan/nsag003 (PMC12948933; doi:10.1093/scan/nsag003)
Supplement: nsag003_Supplementary_Data [file nsag003_supplementary_data.docx]

**Supplementary Materials for**

***Social value orientation modulates fairness decision-making in empathic contexts: Evidence from event-related potentials and neural oscillations***

## **Measurement Scales**

**Assessment of** **trait empathy.** The Chinese version of Davis' (1983) IRI scale was used to measure the participants' levels of trait empathy (Rong et al., 2010). The scale includes four subscales: perspective taking (PT), empathic concern (EC), fantasy (FS), and personal distress (PD). The sum of the cognitive and emotional empathy scores is the total empathy score.

**Assessment of state empathy.** The Empathic Response Scale was used to assess participants’ state empathy levels after selecting the beneficiary. The scale comprised three 7-point items measuring the extent to which participants felt sympathetic, concerned, and touched (Pfattheicher et al., 2019), with the average score of these items taken as the state empathy score.

**Manipulation of State Empathy**

At the beginning of each block of the third-party decision task, the participant’s empathy state was manipulated and checked. Each participant performed the task six times, making decisions on behalf of six different beneficiaries: three in need of help (empathy condition) and three not in need (non-empathy condition).

**Empathy Condition:** Participants were required to choose a beneficiary from a list of public welfare projects and make decisions for them in the third-party decision task. The projects were sourced from Alipay’s official public welfare platform (https://love.alipay.com/donate/index.htm). The following are the potential project names:

1. “Assisting Early Recovery for Critical Illness”

2. “Supporting Left-behind Children in Tibetan Areas”

3. “Assistance for Orphaned Girls”

4. “Warmhearted Care for the Disabled Elderly”

5. “Support for Women in Need”

6. “Skills Training for Older Adults”

7. “Nutrition Improvement for Babies in Poverty”

8. “Guardianship for Isolated Elderly”

**Non-empathy condition.** In this condition, participants selected a beneficiary from four phone numbers linked to individuals who had previously participated in unrelated studies. The possible phone numbers were:

1. 18634614406

2. 18910536405

3. 18643687709

4. 13904360646

5. 15638580593

6. 17719553857

7. 15701924768

8. 18806337721

**Overview of Supplementary Analyses**

The primary analyses reported in the main text focused on the ERP results. Here, we present detailed ANOVA results of behavioral data and subjective ratings, along with supporting PCA-ERP evidence and correlation analyses between behavioral and neural measures.

***Assessment of Trait Empathy and State Empathy***

**Trait Empathy.** The results of the independent samples t-test showed that there was no significant difference between the prosocial group and the proself group in the scores for each dimension of the IRI (PT, EC, FS, PD), as well as in the total score (*p*s > 0.1), indicating that the two groups were similar in terms of trait empathy.

**State Empathy.** A repeated measures ANOVA was conducted on the state empathy scores, with SVO type as the between-subjects factor and empathy induction as the within-subjects factor. The main effect of empathy induction was significant, *F*(1,56) = 254.55, *p* < 0.001, η_p_^2^ = 0.820, with state empathy scores significantly higher in the empathy condition (5.93 ± 0.11) compared to the non-empathy condition (3.13 ± 0.18), indicating that the context that the recipients were individuals in need of help successfully induced state empathy in participants. The main effect of SVO type and the interaction effect between SVO type and empathy induction were not significant (*p*s > 0.1).

***Behavioral Results***

**Acceptance Rates.** An ANOVA on acceptance rates (ARs) revealed significant main effects of empathy induction, *F*(1,56) = 25.76, *p* < 0.001, η_p_^2^ = 0.32, and fairness, *F*(2,112) = 164.49, *p* < 0.001, η_p_^2^ = 0.75. Significant interactions were also observed between empathy induction and fairness, *F*(2,112) = 38.67, *p* < 0.001, η_p_^2^ = 0.41, and among SVO type, empathy induction, and fairness, *F*(2,112) = 5.91, *p* = 0.018, η_p_^2^ = 0.10.

To further explore the interaction between empathy induction and fairness, simple effects analyses were conducted. The results showed that in the non-empathy condition, ARs were significantly higher for the equality offer (0.95) than both the disadvantageous inequality (0.27, *p* < 0.001) and advantageous inequality offers (0.58, *p* < 0.001), with the advantageous inequality offer also significantly higher than the disadvantageous inequality offer (*p* < 0.001). In contrast, in the empathy condition, ARs were significantly higher for the advantageous inequality offer (0.96) than the EQ offer (0.90, *p* = 0.04), while the EQ offer was significantly higher than the disadvantageous inequality offer (0.35, *p* < 0.001).

Additionally, a simple effects analysis was conducted to investigate the three-way interaction among SVO type, empathy induction, and fairness. The results (**Figure 2A**) showed that for the disadvantageous inequality offer, the prosocial group had significantly higher ARs in the empathy condition compared to the non-empathy condition (0.45 vs. 0.28, *p* = 0.011), with no significant difference observed for the proself group. For the EQ offer, ARs were similar in both empathy and non-empathy conditions, regardless of SVO type. Lastly, for the advantageous inequality offer, ARs were significantly higher in the empathy condition compared to the non-empathy condition for both the prosocial (0.97 vs. 0.66, *p* < 0.001) and the proself groups (0.96 vs. 0.50, *p* < 0.001).

**Reaction Time.** An ANOVA on RT data revealed a significant main effect of fairness, *F*(2,112) = 18.61, *p* < 0.001, η_p_^2^ = 0.25, as well as a significant interaction between empathy induction and fairness, *F*(2,112) = 9.62, *p* = 0.003, η_p_^2^ = 0.15. To investigate this two-way interaction, a simple effects analysis was conducted. The results (**Figure 2B**) showed that, in the non-empathy condition, RTs were significantly shorter for the EQ offer (743 ms) compared to both the disadvantageous inequality offer (1082 ms, *p* < 0.001) and the advantageous inequality offer (995 ms, *p* < 0.001), with no difference between the disadvantageous inequality and advantageous inequality offers. Conversely, in the empathy condition, no differences were observed among the three types of offers.

***Subjective Ratings***

**Emotional ratings.** An ANOVA on emotional ratings revealed significant main effects of SVO type, *F*(1,56) = 4.41, *p* = 0.04, η_p_^2^ = 0.07, empathy induction, *F*(1,56) = 10.66, *p* = 0.002, η_p_^2^ = 0.16, and fairness, *F*(2,112) = 225.28, *p* < 0.001, η_p_^2^ = 0.80. Significant two-way interactions were also observed between fairness and SVO type, *F*(2,112) = 8.57, *p* = 0.005, η_p_^2^ = 0.13, and between empathy induction and fairness, *F*(2,112) = 102.80, *p* < 0.001, η_p_^2^ = 0.65. To explore the interaction between empathy induction and fairness, simple effects analyses were conducted. The results (**Figure 2C**) showed that, in the non-empathy condition, emotional ratings were significantly higher for the equality offer compared to both the advantageous inequality offer (*p* = 0.003) and the disadvantageous inequality offer (*p* < 0.001), with the advantageous inequality offer also being rated significantly higher than the disadvantageous inequality offer (*p* < 0.001). In the empathy condition, however, emotional ratings were significantly higher for the advantageous inequality offer compared to the equality offer (*p* < 0.001), and the equality offer was rated significantly higher than the disadvantageous inequality offer (*p* < 0.001).

**Fairness ratings.** An ANOVA on fairness ratings data revealed significant main effects of SVO type, *F*(1,56) = 4.75, *p* = 0.03, η_p_^2^=0.08, empathy induction, *F*(1,56) = 16.76, *p* < 0.001, η_p_^2^ = 0.23, and fairness, *F*(2,112) = 173.18, *p* < 0.001, η_p_^2^ = 0.76. Additionally, significant two-way interactions were observed between SVO type and fairness, *F*(2,112) = 5.58, *p* = 0.02, η_p_^2^ = 0.09, as well as between empathy induction and fairness, *F*(2,112) = 140.89, *p* < 0.001, η_p_^2^ = 0.72. To further explore the interaction between empathy induction and fairness, simple effects analyses were conducted. The results (**Figure 2D**) showed that, in the non-empathy condition, fairness ratings were significantly higher for the equality offer compared to both the advantageous inequality offer (*p* < 0.001) and disadvantageous inequality offer (*p* < 0.001), with the advantageous inequality offer also receiving significantly higher ratings than the disadvantageous inequality offer (*p* < 0.001). In contrast, in the empathy condition, fairness ratings were significantly higher for the advantageous inequality offer than the equality offer (*p* < 0.001), but the equality offer still received significantly higher ratings than the disadvantageous inequality offer (*p* < 0.001).

## **PCA analysis**

To address potential overlap between ERP components, principal component analysis (PCA) was employed to decompose the ERP components (Foti et al., 2011). Time-domain PCA was performed using the Evoked ERP/EPO toolbox in Matlab (Zhang et al., 2020). First, PCA with Promax rotation was conducted on the averaged ERPs at all time points for each participant. Next, the components of interest were identified based on three criteria and mapped onto the electrode field: (1) the polarity and latency of the temporal components; (2) the polarity and spatial distribution of the activated regions; (3) the similarity of the topographical maps of the components of interest across participants and conditions. Finally, the N1 (minimum peak at frontal electrodes 100–200 ms), MFN (mean amplitude at frontal electrodes 300–370 ms), and P3 (mean amplitude at centro-parietal electrodes 410–450 ms) components were chosen for statistical analysis.

To confirm the identification of the PCA factors, Pearson correlations were computed between the amplitudes of PCA factors and the corresponding ERP components derived from traditional time-domain analysis. The results indicated significant correlations between N1 and PCA-N1 (*r* = 0.98, *p* < 0.001), MFN and PCA-MFN (*r* = 0.95, *p* < 0.001), and P3 and PCA-P3 (*r* = 0.99, *p* < 0.001).

## **Time-domain PCA results**

The average waveforms of the PCA-N1, PCA-MFN and PCA-P3 in the non-empathy and empathy conditions for the prosocial and proself groups are shown in Figure S1.


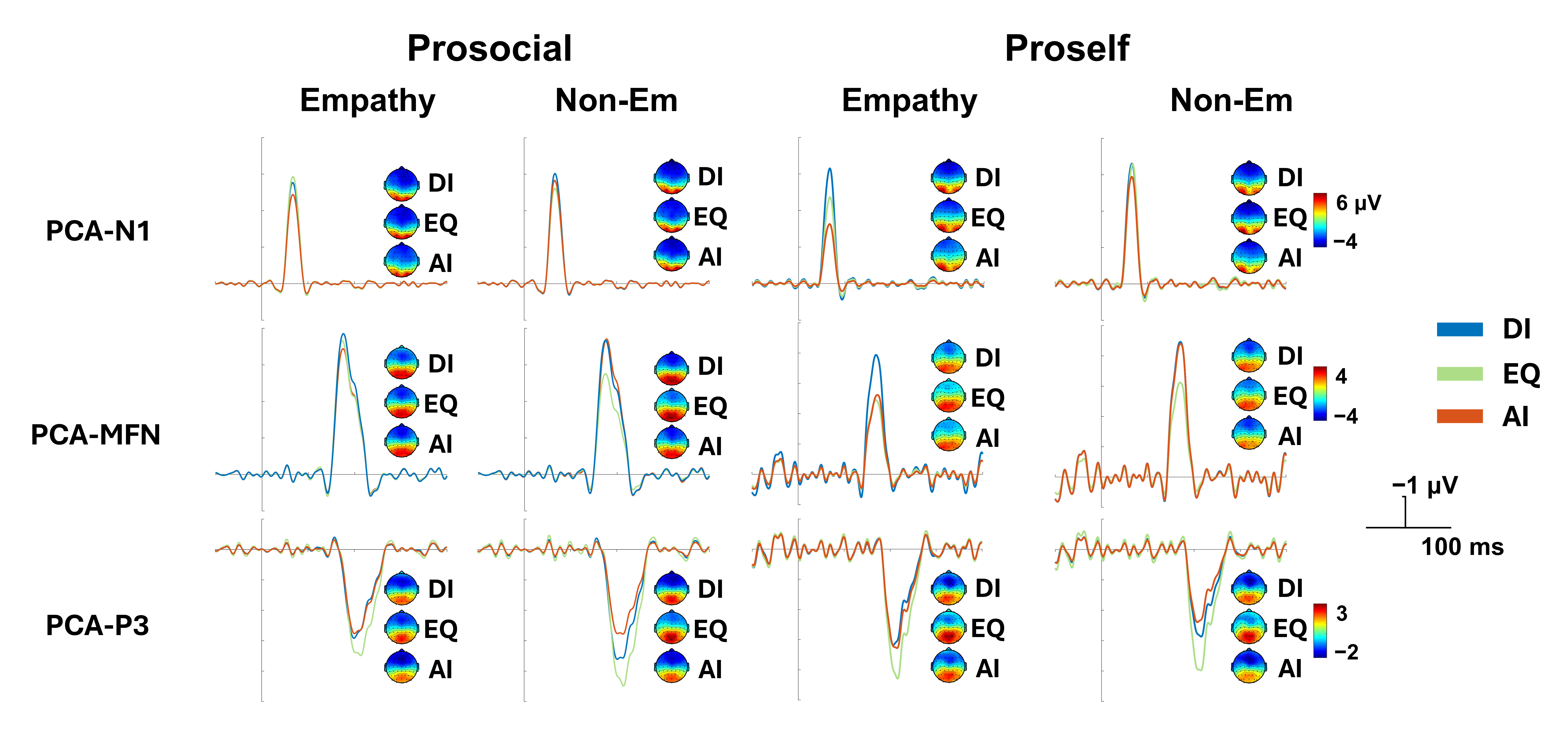


**Figure S1.** The average waveforms of the PCA-N1, PCA-MFN and PCA-P3. (DI: *Disadvantageous inequality*; EQ: *Equality*; AI: *Advantageous inequality*; Non-Em: *Non-empathy*)

**PCA-N1.** An ANOVA on N1 amplitude revealed marginally significant main effect of empathy induction, *F*(1,56) = 3.15, *p* = 0.08, η_p_^2^ = 0.05, and significant main effect of fairness, *F*(2,112) = 9.31, *p* = 0.003, η_p_^2^ = 0.14. Marginally significant interactions were observed among empathy induction, fairness, and SVO type, *F*(2,112) = 2.82, *p* = 0.09, η_p_^2^ = 0.13.

A simple effects analysis was conducted to investigate the three-way interaction among SVO type, empathy induction, and fairness. The results showed that for the DI offer, N1 amplitude was similar between the empathy and non-empathy conditions in both the prosocial and proself groups. For the EQ and AI offer, N1 amplitude was significantly more negative in the non-empathy condition than in the empathy condition for the proself group (EQ: -2.91 μV vs. -1.96 μV, *p* = 0.03; AI: -2.37 μV vs. -1.49 μV, *p* = 0.05), whereas prosocial individuals exhibited no significant difference in N1 amplitude between empathy and non-empathy conditions.

**PCA-MFN.** An ANOVA on MFN amplitude revealed a significant main effect of fairness, *F*(2,112) = 12.02, *p* = 0.001, η_p_^2^ = 0.18, as well as significant two-way interactions between empathy induction and SVO type, *F*(2,112) = 5.02, *p* = 0.03, ηp² = 0.08, between fairness and SVO type, *F*(2,112) = 3.97, *p* = 0.05, η_p_^2^ = 0.07, and between empathy induction and fairness, *F*(2,112) = 2.83, *p* = 0.09, η_p_^2^ = 0.05. The three-way interaction (SVO type × empathy induction × fairness) was not statistically significant.

Given our a priori interest in SVO, we additionally conducted exploratory within-group empathy induction × fairness simple-effects analyses. In the non-empathy condition, unequal offers (both disadvantageous and advantageous inequality) elicited significantly more negative MFN amplitudes than equal offers in both groups. This was shown by comparing equality with disadvantageous and advantageous inequality in both the prosocial (-1.79 vs. -2.61 μV, *p* = 0.047; -1.79 vs. -2.68 μV, *p* = 0.037) and proself groups (-2.55 vs. -3.65 μV, *p* = 0.014; 2.55 vs. -3.58 μV, *p* = 0.025). In the empathy condition, the proself group displayed significantly enhanced MFN for DI offers (-3.23 μV) compared to both EQ offer (-2.01 μV, *p* = 0.001) and AI offer (-2.13 μV, *p* = 0.01), whereas no difference was observed among the three types offer for the prosocial group.

**PCA-P3.** An ANOVA on P3 amplitude revealed a significant main effect of fairness, *F*(2,112) = 26.45, *p* < 0.001, η_p_^2^ = 0.32, and a significant interaction between empathy induction and SVO type, *F*(1,56) = 4.39, *p* = 0.04, η_p_^2^ = 0.07. To further explore the interaction between empathy induction and SVO type, simple effects analyses were conducted. The results showed that proself individuals did not show a significant difference in P3 amplitude between empathy and non-empathy conditions (*p* ≥ 0.1). In contrast, prosocial individuals showed a marginally significant reduction in P3 amplitude under empathy induction (1.89 μV) compared to the non-empathy condition (2.51 μV), *p* = 0.07.

## **Correlation analysis**

**Table S1.** Correlation results between SVO and the acceptance rates of DI and AI offers, as well as ERP/ERO component amplitude/power difference in the non-empathy condition.

|  | SVO | DI AR | AI AR | dN1 | aN1 | dMFN | aMFN | dP3 | aP3 | dAlpha |
| --- | --- | --- | --- | --- | --- | --- | --- | --- | --- | --- |
| DI AR | 0.022 |  |  |  |  |  |  |  |  |  |
| AI AR | 0.197 | 0.504^***^ |  |  |  |  |  |  |  |  |
| dN1 | 0.120 | -0.095 | 0.124 |  |  |  |  |  |  |  |
| aN1 | 0.211 | 0.101 | 0.129 | 0.323^**^ |  |  |  |  |  |  |
| dMFN | 0.008 | -0.064 | 0.084 | 0.630^***^ | 0.043 |  |  |  |  |  |
| aMFN | 0.022 | 0.103 | -0.010 | 0.256^*^ | 0.315^**^ | 0.526^***^ |  |  |  |  |
| dP3 | 0.021 | -0.151 | -0.156 | 0.383^**^ | -0.043 | 0.753^***^ | 0.358^**^ |  |  |  |
| aP3 | 0.063 | -0.055 | -0.247^*^ | 0.170 | 0.351^**^ | 0.309^**^ | 0.772^***^ | 0.426^***^ |  |  |
| dAlpha | 0.187 | 0.182 | 0.110 | 0.137 | -0.085 | -0.005 | -0.171 | -0.071 | -0.169 |  |
| aAlpha | -0.113 | 0.142 | 0.011 | 0.228^*^ | -0.250^*^ | 0.151 | -0.009 | 0.070 | -0.112 | 0.467^***^ |

Note: **p* < 0.05, ***p* < 0.01, ****p* < 0.001. (**DI AR**: *Acceptance rate for disadvantageous inequality offers*; **AI AR**: *Acceptance rate for advantageous inequality offers*; **dERP/ERO component**: *ERP/ERO component amplitude/power difference between equality and disadvantageous inequality offers*; **aERP/ERO component**: *ERP/ERO component amplitude/power difference between equality and advantageous inequality offers*; the same below)

**Table S2.** Correlation results between SVO and the acceptance rates of DI and AI offers, as well as ERP/ERO component amplitude/power difference in the empathy condition

|  | SVO | DI AR | AI AR | dN1 | aN1 | dMFN | aMFN | dP3 | aP3 | dAlpha |
| --- | --- | --- | --- | --- | --- | --- | --- | --- | --- | --- |
| DI AR | 0.247^*^ |  |  |  |  |  |  |  |  |  |
| AI AR | 0.064 | 0.197 |  |  |  |  |  |  |  |  |
| dN1 | -0.132 | -0.194 | 0.023 |  |  |  |  |  |  |  |
| aN1 | 0.072 | -0.092 | -0.078 | 0.663^***^ |  |  |  |  |  |  |
| dMFN | -0.264^*^ | -0.335^**^ | -0.278^*^ | 0.494^***^ | 0.509^***^ |  |  |  |  |  |
| aMFN | 0.016 | -0.107 | -0.211 | 0.270^*^ | 0.628^***^ | 0.595^***^ |  |  |  |  |
| dP3 | -0.124 | -0.201 | -0.249^*^ | 0.351^**^ | 0.346^**^ | 0.652^***^ | 0.328^**^ |  |  |  |
| aP3 | -0.052 | -0.034 | -0.267^*^ | 0.274^*^ | 0.515^***^ | 0.545^***^ | 0.775^***^ | 0.635^***^ |  |  |
| dAlpha | 0.105 | -0.298^*^ | -0.165 | 0.105 | 0.344^**^ | 0.197 | 0.299^*^ | 0.308^**^ | 0.318^**^ |  |
| aAlpha | 0.159 | -0.300^*^ | -0.147 | -0.048 | 0.188 | 0.169 | 0.162 | 0.249^*^ | 0.148 | 0.821^***^ |

Note: **p* < 0.05, ***p* < 0.01, ****p* < 0.001.
